# A Strategy Inspired by the Cicada Shedding Its Skin for Synthesizing the Natural Material NaFe_3_S_5_·2H_2_O

**DOI:** 10.1002/advs.202301324

**Published:** 2023-05-10

**Authors:** Hanqing Dai, Wenqing Dai, Yuanyuan Chen, Yukun Yan, Guangzheng Zuo, Zhe Hu, Jinxin Wei, Wenjie Zhou, Wanlu Zhang, Wei Wei, Guoqi Zhang, Ruiqian Guo

**Affiliations:** ^1^ Academy for Engineering and Technology Fudan University Shanghai 200433 China; ^2^ School of Materials Science and Engineering Shanghai Jiao Tong University Shanghai 200240 China; ^3^ Institute for Electric Light Sources Fudan University Shanghai 200433 China; ^4^ College of Electronic and Optical Engineering Nanjing University of Posts and Telecommunications Nanjing 210023 China; ^5^ Electronic Components Technology and Materials Delft University of Technology Delft 2628 CD Netherlands

**Keywords:** catalyze, energy storage, NaFe_3_S_5_·2H_2_O, sulfide mineral

## Abstract

Sulfide minerals hold significant importance in both fundamental science and industrial advancement. However, certain natural sulfide minerals, such as NaFe_3_S_5_·2H_2_O (NFS), pose great challenges for exploitation and synthesis due to their high susceptibility to oxidation. To date, no successful precedent exists for synthesizing NFS. Here, a novel approach to synthesizing low‐cost and pollution‐free NFS with high stability using the high‐pressure hydrothermal method based solely on knowledge of its chemical formula is presented. Moreover, an innovative strategy inspired by the cicada's molting process to develop unstable natural materials is proposed. The mechanical, thermal, optical, electrochemical, and magnetic properties of the NFS are thoroughly investigated. The storage of lithium, sodium, and potassium ions is primarily concentrated in the gap between (0 0 1) crystal planes. Additionally, as a catalyst for hydrogen evolution reaction (HER) at 10 mA cm^−2^, micron‐sized NFS exhibits an excellent overpotential of 6.5 mV at 90 °C, surpassing those of reported HER catalysts of similar size. This research bridges the gap in the sulfide mineral family, overcomes limitations of the high‐pressure hydrothermal method, and paves the way for future synthesis of natural minerals, lunar minerals, and Martian minerals.

## Graphical abstract

In this work, a systematic approach for synthesizing NaFe_3_S_5_·2H_2_O (NFS) with water–oxygen stability by the high‐pressure hydrothermal method is presented. An analogy to cicada molting as a strategy for preparing unstable natural materials in water–oxygen conditions is also proposed. The limitations of the high‐pressure hydrothermal method are broken through, which hold significant scientific implications for its potential application in synthesizing lunar and Martian minerals. These works provide fundamental data of the NFS and a general route to synthesize newly discovered minerals based solely on their chemical formula. This opens up new possibilities for achieving novel applications and producing inexpensive three‐dimensional inorganic catalysts. Ultimately, the reduced cost renders the three‐dimensional NFS catalysts more appealing for practical applications.

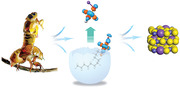

## 1 Introduction

Sulfide^[^ ^1^ ^]^ minerals (for example, CoFeS_2_,^[^ ^2^, ^3^ ^]^ CuFeS_2_,^[^ ^4^, ^5^ ^]^ NiFeS_2_,^[^ ^6^, ^7^ ^]^ AuFeS_2_,^[^ ^8^ ^]^ NaFeS_2_,^[^ ^9^, ^10^ ^]^ FeS_2_,^[^ ^11^, ^12^ ^]^ MoS_2_,^[^ ^13^, ^14^ ^]^ and others) are widely applied in various fields such as photodetectors,^[^ ^13^, ^14^, ^15^, ^16^, ^17^ ^]^ sensors,^[^ ^18^, ^19^, ^20^ ^]^ energy storage,^[^ ^14^, ^21^, ^22^ ^]^ catalysis,^[^ ^23^, ^24^, ^25^ ^]^ wearable devices,^[^ ^26^, ^27^, ^28^ ^]^ antibiosis,^[^ ^29^, ^30^, ^31^ ^]^ and so forth. This is due to their exceptional properties such as tunable band gap, good catalytic activity, high melting temperature, excellent ionic intercalation and thermoelectric performance. As a novel member of this family, the natural NFS was discovered by Richard C. Erd and Gerald K. Czamanske in 1983. However, only a few milligrams have been located thus far. Unfortunately, this natural NFS is highly unstable in water–oxygen environments^[^ ^1^, ^32^, ^33^ ^]^ and has yet to be synthesized since its advent. Consequently, its properties, structure and potential applications remain shrouded in mystery.

**Figure 1:**
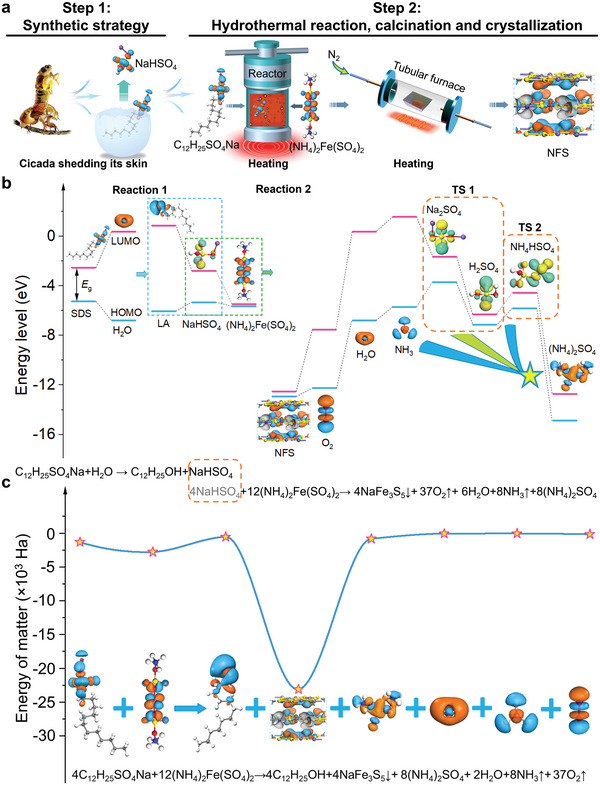
Synthesis route and reaction mechanisms of the NaFe_3_S_5_·2H_2_O. a) Schematic of the NaFe_3_S_5_·2H_2_O preparation. The dried red powders were calcined under nitrogen conditions to obtain NaFe_3_S_5_·2H_2_O crystals covered with carbon layer. b) Frontier molecular orbital simulation. The distributions of the highest occupied molecular orbital (HOMO) and lowest unoccupied molecular orbital (LUMO) with their energy gaps (*E*_g_) of reactants and possible products were calculated by density functional theory (DFT) simulation with the m‐GGA M06‐L level. Moreover, energies of reactants and products were calculated by the DFT simulation are displayed in Figure S1 (Supporting Information). And it should be noted that in inorganic materials, the HOMO bears similarity to the valence band while the LUMO is akin to the conduttion band. According to the second law of thermodynamics, any isothermal and isobaric closed system tends to reduce the Gibbs free energy. Therefore, the analysis of enthalpy (the difference in energy between reactants and products) in the system can deduce possible chemical reaction processes. These results illustrated that the reaction processes of the NFS generation include reaction (1) (C_12_H_25_SO_4_Na + H_2_O → C_12_H_25_OH + NaHSO_4_) and reaction (2) (4NaHSO_4_ + 12(NH_4_)_2_Fe(SO_4_)_2_→4NaFe_3_S_5_↓ + 8(NH_4_)_2_SO_4_ + 6H_2_O + 8NH_3_↑ + 37O_2_↑). And reaction (2) may contain two transitional reactions: (TS 1) 12(NH_4_)_2_Fe(SO_4_)_2_ + 8NaHSO_4_ → 4NaFe_3_S_5_↓ + 10H_2_SO_4_ + 2Na_2_SO_4_ + 6H_2_O + 24NH_3_↑ + 37O_2_↑; (TS 2) 12(NH_4_)_2_Fe(SO_4_)_2_ + 4NaHSO_4_ → 4NaFe_3_S_5_↓ + 8NH_4_HSO_4_ + 6H_2_O + 16NH_3_↑ + 37O_2_↑. c) Energies of reactants and products were calculated by the DFT simulation. Reaction (1) is endothermic reaction, reaction (2) is exothermic reaction, and the total reaction is exothermic reaction.

It is widely acknowledged that high‐temperature and high‐pressure synthesis represents a potent approach for generating novel materials. However, this method is unsuitable for synthesizing the NFS with unknown structure, nary precedent, and extreme instability in water–oxygen environments. In the embodiment of synthesizing the NFS, we have devised a strategy akin to the cicada shedding its skin. We utilized hydrophilic surfactants as indirect reactants to protect natural NFS while simultaneously releasing direct reactants, so that the stable NFS was successfully synthesized in water–oxygen environments using the high‐pressure hydrothermal method (**Figure** **1**a).

## 2 Results and Discussion

In this embodiment, the initial step involves obtaining the structure of NFS to identify the reactants. However, the crystallographic data source^[^ ^1^ ^]^ of the NFS is antiquated and its accuracy remains unknown; thus far, its crystal structure has not been resolved. Hence, we sought a substance with a similar chemical structure and inferred an NFS‐like configuration. Firstly, it was evident that sulfur elements should be searched. Secondly, any such substance possessing an analogous NFS structure would contain IA group elements. Surprisingly, a comparable substance (RbBi_3_S_5_) was promptly discovered in the Crystallography Open Database. Ultimately, an inferred structure of the NaFe_3_S_5_ denoting the NFS was simulated through element replacement (Figure 1a).

The second step involves identifying potential reactants based on the assumption that the inferred structure is correct. Initially, (NH_4_)_2_Fe(SO_4_)_2_ was tentatively chosen as one of the initial reactants based on the redox characteristics of sulfide minerals and the elementary composition of NFS. However, determining other initial reactants providing sodium or sulfur elements presents a primary challenge. Furthermore, by calculating the energies of ionized ions of (NH_4_)_2_Fe(SO_4_)_2_ and the population distribution of NFS, we were surprised to find that NaHSO_4_ providing sodium elements is a core reactant. However, NaHSO_4_ cannot directly participate in the reaction. Taking inspiration from the cicada's molting process, we proposed a strategy for liberating NaHSO_4_ from certain substances and facilitating its immediate participation in the reaction. Here, the selection of the NaHSO_4_ release source is a secondary challenge. While, NFS is extremely easy to oxidize in the process of formation, and making the generated NFS stable in the solution poses a tertiary obstacle. Based on these ionized ions (Na^+^, H^+^, SO_4_^2−^) of NaHSO_4_, as well as considerations of organic matter water‐solubility and the electronic density of states calculations for NFS, it is preferable for hydrophilic anionic surfactants to participate in the reaction and protect NFS. After analyzing of energy levels and ionization energies of various hydrophilic anionic surfactants, (C_12_H_25_SO_4_Na) may be selected as a suitable source of NaHSO_4_.

(1) $$C_{12}H_{25}{\mathrm{SO}}_{4}\mathrm{Na}+H_{2}O\to C_{12}H_{25}\mathrm{OH}+{\mathrm{NaHSO}}_{4}$$

(2) $$\begin{array}{ccc} 4{\mathrm{NaHSO}}_{4} & + & 12{\left({\mathrm{NH}}_{4}\right)}_{2}\mathrm{Fe}{\left({\mathrm{SO}}_{4}\right)}_{2}\to 4{\mathrm{NaFe}}_{3}S_{5}↓\\ & & +8{\left({\mathrm{NH}}_{4}\right)}_{2}{\mathrm{SO}}_{4}\;+6H_{2}O+8{\mathrm{NH}}_{3}↑+37O_{2}↑ \end{array}$$

Thirdly, (NH_4_)_2_Fe(SO_4_)_2_, C_12_H_25_SO_4_Na, and H_2_O were selected as the initial reactants for the experiment based on the aforementioned considerations. The potential products encompass C_12_H_25_OH (LA), NaHSO_4_, NaFe_3_S_5_, (NH_4_)_2_SO_4_, H_2_SO_4_, Na_2_SO_4_, NH_4_HSO_4_, H_2_O, NH_3_, and O_2_. Happily, NFS was successfully synthesized and remained stable in the solution. By analyzing the reaction process, it was discovered that C_12_H_25_SO_4_Na (analogous to the cicada in its shell) released NaHSO_4_ (an analogue to a shelled cicada) and generated LA (similar to the sloughed skin of a cicada) at high temperature (Equation (1)). The reaction process is analogous to the cicada shedding its exoskeleton (Figure 1a). By cleverly utilizing the protection of LA and unreacted C_12_H_25_SO_4_Na, NaHSO_4_ (analogous to the shelled cicada) can react with (NH_4_)_2_Fe(SO_4_)_2_ to form stable NFS in water–oxygen environments (Equation (2)). The crucial reason is that unreacted C_12_H_25_SO_4_Na can instantaneously adhere to the newly generated NFS surface and protect NFS together with the LA during the reaction process. Finally, the density functional theory (DFT) calculations confirm the rationality of NFS generation reaction processes (Figure 1b,c and Figure S1, Supporting Information, *detailed in the annotation section of* Figure 1).

**Figure 2:**
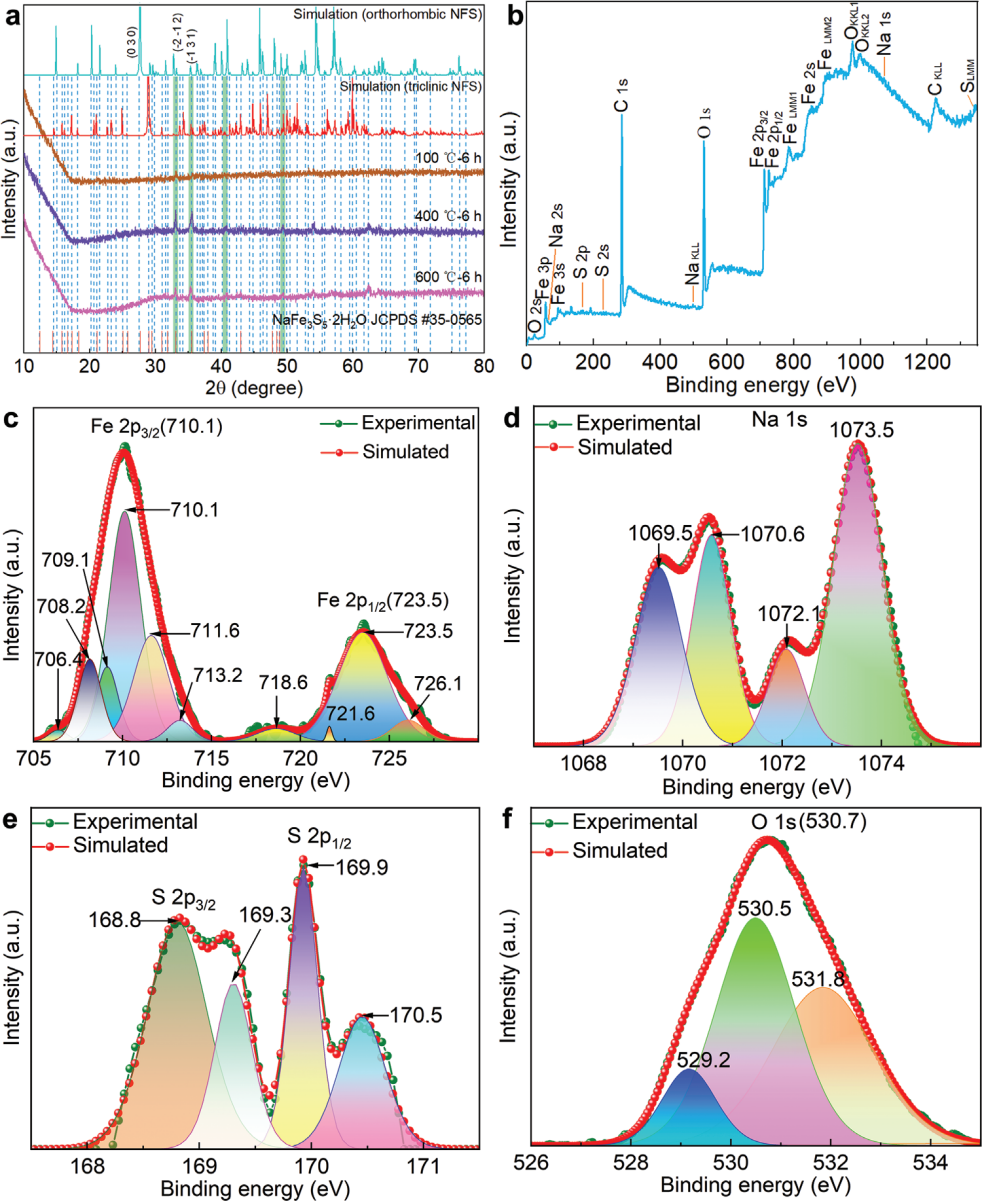
The structure of the NaFe_3_S_5_·2H_2_O. a) XRD pattern of the simulated orthorhombic and triclinic NaFe_3_S_5_ as well as annealed NaFe_3_S_5_·2H_2_O at temperatures of 100, 400, and 600 °C for 6 h respectively. b) The survey XPS spectrum of the NaFe_3_S_5_·2H_2_O. c–f) High‐resolution XPS spectrum of Fe 2p, Na 1s, S 2p, and O 1s acquired from the NaFe_3_S_5_·2H_2_O.

To obtain stable crystals, the NFS products were calcined under nitrogen conditions, resulting in the formation of a dense carbon protective layer on the surface of NFS. The X‐ray powder diffraction (XRD) results indicate that a heat treatment temperature of 400 °C is optimal for the crystallization of JCPDS #35‐0565 NaFe_3_S_5_·2H_2_O (**Figure** **2**a), which belongs to the triclinic system, and its lattice parameters are *a* = 7.409 Å, *b* = 9.881 Å,*c* = 6.441 Å, and *α* = *β* = *γ* = 90° respectively. The results are consistent with the reported results.^[^ ^1^ ^]^ To validate the inferred crystal structure of NFS, we simulated two XRD patterns of the inferred orthorhombic and triclinic structures. Based on several obvious peaks (Figure 2a), it can be concluded that NFS treated at 400 and 600 °C tends toward an orthorhombic structure, but NFS treated at 100 °C leans more toward a triclinic structure. However, the NFS still contains some triclinic crystals under 400 °C heat treatment. This phenomenon may be attributed to the fact that certain NFS undergo a conversion process from room temperature to 400 °C, during which they first transform into triclinic crystals and then become resistant to further transformation into orthorhombic crystals. In this work, our research and experiments on the properties of NFS are based on NFS treated at 400 °C. Fortunately, the XRD pattern of the inferred orthorhombic structure of the NaFe_3_S_5_ denoting NFS is simulated through element replacement, which is consistent with the XRD of NFS treated at 400 °C (Figure 2a). These results also suggest that the synthesis of NFS has been successfully achieved. In summary, our research has successfully addressed four challenges encountered in the synthesis of NFS: determining its structure, selecting core reactants, sourcing these reactants and ensuring the stable existence of NFS in solution. Furthermore, our findings offer valuable insights for synthesizing natural minerals based solely on their chemical formula.

Afterward, the composition, chemical, and electronic states of elements in the NFS were measured. A wide XPS survey of the NFS is displayed in Figure 2b, which betokens that the samples contained Na, Fe, S, and O elements, respectively. The high‐resolution XPS spectra of Na 1s, Fe 2p, S 2p, and O 1s are shown in Figure 2c–f, respectively. In Figure 2c, the binding energies of the Fe 2p_3/2_ and Fe 2p_1/2_ peaks are located at 710.1 and 723.5 eV, with a shakeup satellite peak at 718.6 eV, which are characteristic for the Fe^3+^ species.^[^ ^34^, ^35^, ^36^ ^]^ And the fitted energy difference between the Fe 2p_1/2_ and Fe 2p_3/2_ lines is ≈13.4 eV, which slightly coincides with the reference value ∆*E* = 13.6 eV for Fe^3+^.^[^ ^34^, ^35^, ^36^ ^]^ Then, the peaks around 1069.5, 1070.6, 1072.1, and 1073.5 eV are consistent with the ionic bindings of Na element (Figure 2d). The binding energies of the S 2p_3/2_ and S 2p_1/2_ peaks are located at 168.8 and 169.9 eV (Figure 2e). Additionally, the peaks around 529.2, 530.5, and 531.8 eV are consistent with the bindings of O element (Figure 2f). The results suggest that iron oxide is not a possible product and the products are likely to be NFS.

**Figure 3:**
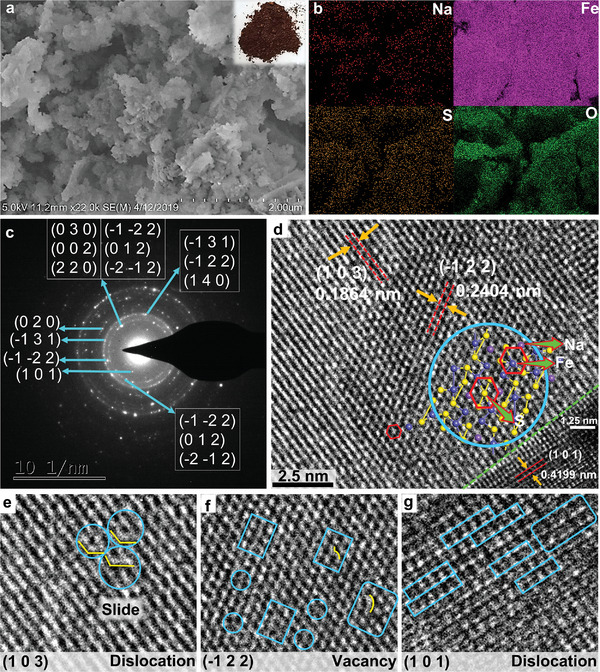
The morphology of the NaFe_3_S_5_·2H_2_O. a) SEM. b) Elemental mapping of Na, Fe, S, and O. c) Electron diffraction pattern. d) HRTEM image. The illustration is the crystal structure of the NaFe_3_S_5_. e) The dislocation of (1 0 3) crystal plane. f) The vacancy of (−1 2 2) crystal plane. g) The dislocation of (1 0 1) crystal plane.

To exclude the possibility of the product being other substances with similar structures to the NFS, the morphology and fine structure of NFS are detected by a scanning electron microscope (SEM) and high‐resolution transmission electron microscope (HRTEM) respectively. The SEM image provides that these products are mainly three‐dimensional flocculus with rare flakes (**Figure** **3**a). Moreover, the imaging capabilities of SEM are combined with an energy dispersive X‐ray spectrometer (EDS) to achieve X‐ray mapping of elemental distributions of the product for characterizing microstructures, which implies the distribution of Na, Fe, S, and O on the surface of the product (Figure 3b). Besides, the HRTEM image displays that the diffraction spots are scattered while a faint annular outline is discernible, indicating that the products are polycrystalline and contain many grains (Figure 3c). According to the comparison of the distances between the multiple crystal planes (Figure 3c,d), we preliminarily confirm that it is highly consistent with the reported results.^[^ ^1^ ^]^ Its crystal structure is constructed by DFT and may be matched with HRTEM image, as displayed in the illustration of Figure 3d. The results of HRTEM reveal that there are dislocations in (1 0 3) (Figure 3e) and (1 0 1) (Figure 3g) crystal planes, and there are vacancies in the (−1 2 2) (Figure 3f) crystal plane.

By analyzing the HRTEM and XRD of NFS, it is speculated that the near disappearance of the (0 3 0) crystal peak might be caused by the proximity of (−1 −2 2), (0 1 2), (−2 −1 2), (0 0 2), (2 2 0), and (0 3 0) planes or the crystal‐plane slip of (0 0 1) (Figures 2a and 3c). The enhancement of (−2 −1 2) crystal peaks may be due to the proximity of (−1 −2 2), (0 1 2), and (−2 −1 2) planes (Figures 2a and 3c). The enhancement of the (−1 3 1) crystal peak may be due to the proximity of (−1 2 2) and (1 0 4) planes (Figures 2a and 3c). Based on HRTEM, XRD and simulations, it can be considered that the experimental and simulations of the XRD are highly matched with the standard card, and the possibility of other substances possessing similar structures to NFS is eliminated. Consequently, these results strongly support the rationality of our constructed crystal structure and validate the successful synthesis of NFS.

Here, the band structures and partial density of states (PDOS) of the NaFe_3_S_5_ (denoting the NFS) with Na, Fe, and S were investigated respectively (Figure S2, Supporting Information). The calculated band structures confirmed that the NFS is an excellent conductor (Figure S2a, Supporting Information). And the angular momentum (*l*‐dependent) origin of the various bands is identifiable from the PDOS (Figure S2b, Supporting Information). The lowest energy group around −52.1 eV has mainly a Na‐s state (Figure S2b,c, Supporting Information). The second group around −24.0 eV has significant contributions from Na‐p with a small contribution of S‐p state (Figure S2b,c,e, Supporting Information). The deeper sub‐band group from −19.7 to −10.7 eV originates from S‐s/p and Fe‐s states with a small contribution of Fe‐p/d states (Figure S2b,d,e, Supporting Information). The groups from −10.7 eV up to Fermi energy (*E*_F_) originate from S‐p and Fe‐d states with a small contribution of S‐s, Fe‐s/p, and Na‐s/p states (Figure S2b–e, Supporting Information). The groups from *E*_F_ to 2.1 eV are mainly of S‐p and Fe‐d states with a small contribution of S‐s and Fe‐s/p states (Figure S2b,d,e, Supporting Information). The groups above 2.1 eV are mainly of S‐p, Fe‐p, and Na‐p states (Figure S2b–e, Supporting Information). Additionally, the difference of charge density for the NFS with (0 0 1) crystal plane is displayed in Figure S2f (Supporting Information). The charge density surrounding the Fe and S atoms exceeds that of the Na atom, while maintaining local charge distribution and structural stability. This indicates that the electronic conductivity in NFS is primarily derived from the Fe and S atoms with fixed positions.

**Figure 4:**
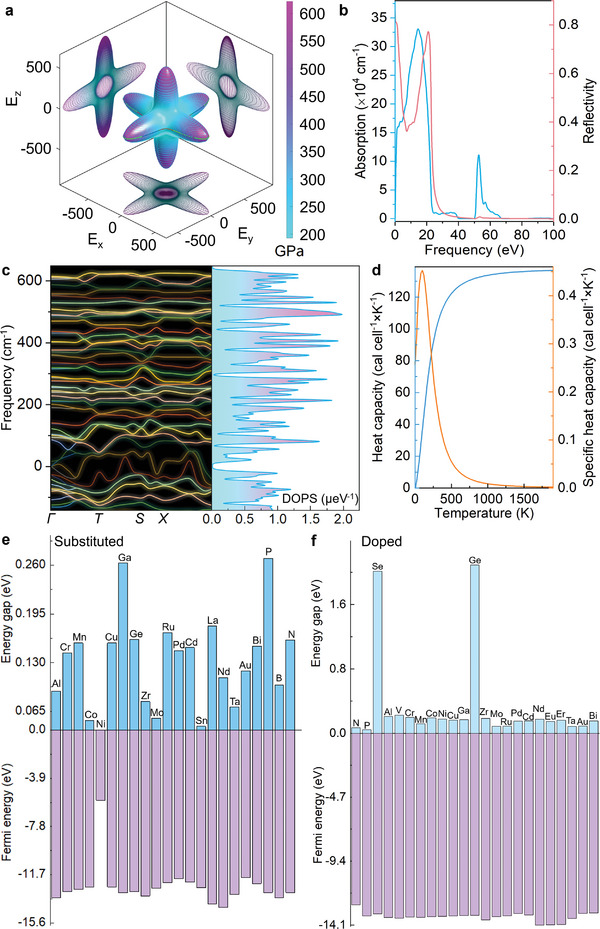
The mechanical, optical and thermoelectric properties as well as tunable band gaps of the NaFe_3_S_5_ crystal model (denoting the NaFe_3_S_5_·2H_2_O). a) Three‐dimensional distribution of Young's modulus. b) Ultraviolet–visible spectroscopy absorption and reflectivity spectra. c) Phonon dispersion curves and phonon density of states (DOPS). d) Heat capacity and specific heat capacity of a NaFe_3_S_5_ cell (*a* = 7.409 Å,*b* = 9.881 Å,*c* = 6.441 Å). Energy gaps and Fermi energies of e) substituted and f) doped NaFe_3_S_5_.

Besides, its magnetic and mechanical properties have been investigated. The magnetization curve suggests the NFS is an important hard magnetic material with a coercive force of 63.5 Oe (Figure S3, Supporting Information), which may have important applications in magnetoelectric measuring instruments, loudspeakers, communication devices, and other related fields. Additionally, the molecular dynamics calculations illustrated that the compressibility of the NFS is about 4.2 TPa^−1^ and the components of Young's modulus in the *x*‐, *y*‐, and *z*‐axes are 622.3, 334.6, and 278.5 GPa respectively (**Figure** **4**a). These results indicate that the NFS is an anisotropic material with higher hardness and elastic modulus than those of RuB_2_, OsB_2_, and ZrB_4_,^[^ ^37^, ^38^ ^]^ and may be used as a novel cement.

Moreover, the DFT calculations have unveiled optical and thermoelectric properties as well as tunable band gaps. The optical results suggest that the NFS is reflective in the far infrared range and exhibits strong absorption in the ultraviolet–visible spectral range (Figure 4b). Thus, the NFS will serve as a special coating material.^[^ ^39^, ^40^, ^41^ ^]^ However, the structure of the NFS exhibits inferior thermodynamic stability, and a few virtual frequencies are observed in Figure 4c. Nevertheless, experimental results show that it can remain stable at temperatures up to 600 °C under conditions where water and oxygen are isolated (Figure 2a). These imply that the NFS may be applied as an indicator in industrial field.^[^ ^42^ ^]^ Additionally, the heat capacity of the NFS is revealed in Figure 4d, and the specific heat capacity is maxed at 105.7 K, which means that the NFS might be used as an anti‐freezing agent.^[^ ^43^, ^44^, ^45^ ^]^ Moreover, the DFT calculations demonstrate that the band gap of NFS can be regulated from 0 to 2.09 eV by element doping and substitution (Figure 4e,f), and the band gaps of NFS‐Ge and NFS‐Se doped are 2.09 and 2.01 eV respectively (Figure 4f). These results indicate that the NFS with a tunable band gap will be widely applied in many fields of photodetectors, sensors, energy storage, catalysis, wearable devices, antibiosis, and others.

**Figure 5:**
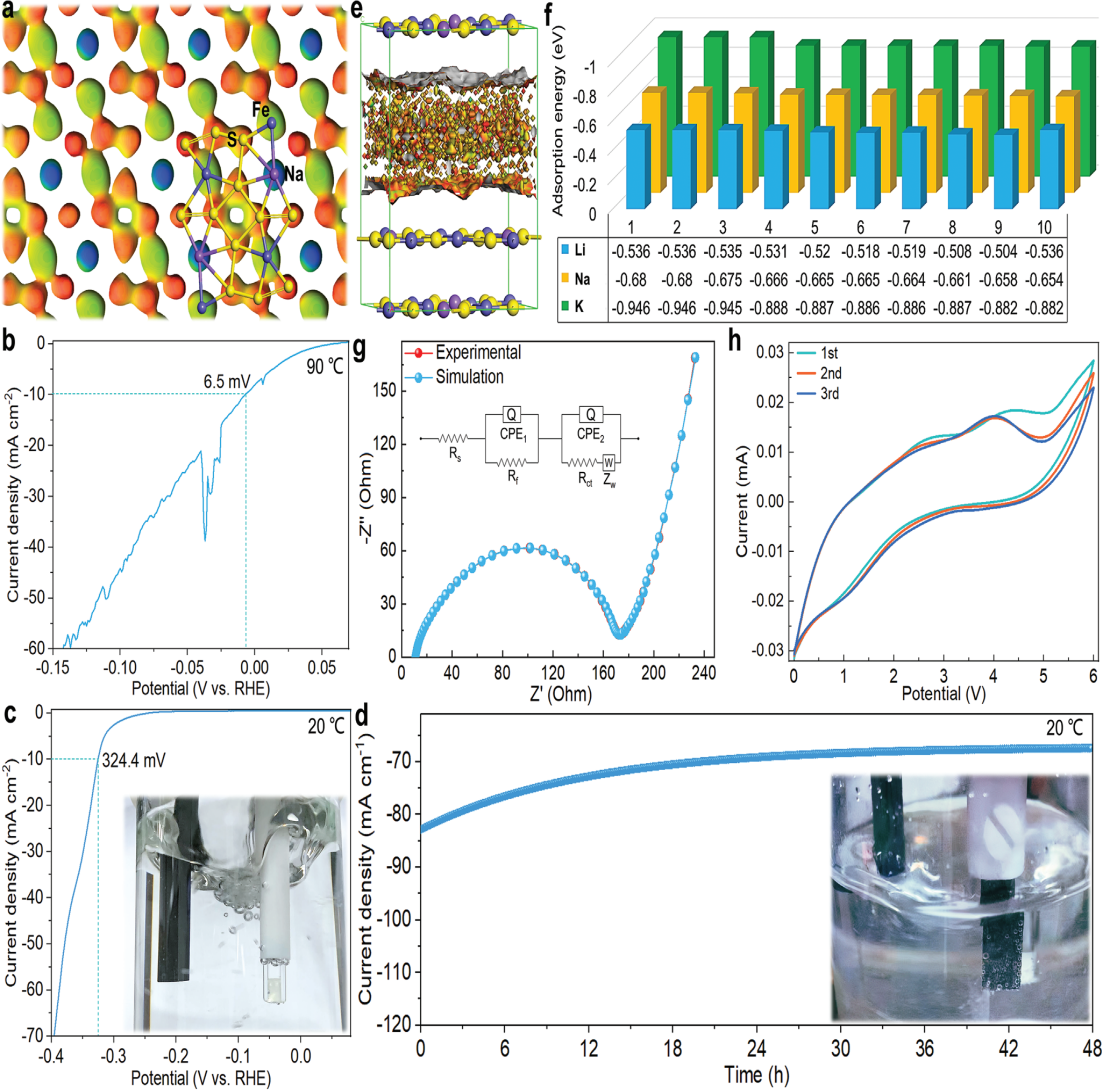
Performance of the NaFe_3_S_5_·2H_2_O. a) The view of the three‐dimensional Fukui function Hirshfeld surface. b) LSV polarization curve for the NaFe_3_S_5_·2H_2_O loaded on a carbon paper (0.5 × 0.5 cm^−2^) at 90 °C. c) LSV polarization curve for the NaFe_3_S_5_·2H_2_O loaded on a glassy carbon electrode at 20 °C, the inset is a photo of catalytic process. d) Long‐term stability of the NaFe_3_S_5_·2H_2_O (loaded on a carbon paper (0.5 × 0.5 cm^−2^) to avoid the NaFe_3_S_5_·2H_2_O falling off) at 500 mV for 48 h with iR‐compensation at 20 °C, the inset is a photo of catalytic process. e) The adsorption regions of ions between (0 0 1) crystal planes shown by the adsorption model. f) The adsorption energies of different adsorption sites for lithium, sodium, and potassium ions in the adsorption regions between (0 0 1) crystal planes. g) Nyquist plots of Na/NFS cell. h) CV curves of NFS electrode at a scan rate of 0.1 V s^−1^ in the potential range of 0.0–6.0 V versus Na/Na^+^.

Furthermore, the Fukui function which resulted from a derivative of electron density keeping the positions of nuclei unchanged is a more practical and convenient way of predicting favorable sites for electrophilic or nucleophilic attack.^[^ ^46^, ^47^, ^48^ ^]^ Here, the Fukui function is borrowed to describe the surface activity of the NFS (**Figure** **5**a and Table S1, Supporting Information). The DFT calculations reflect those atoms of the NFS surface have a susceptibility to electrophilic attack, which means that the NFS can be used as a catalyst for hydrogen evolution reaction (HER) of sustainable industrial‐scale hydrogen generation.

As an example, the detailed results of the linear sweep voltammetry (LSV) curves for the NFS are presented in Figure 5b,c (Video S1, Supporting Information). Although the catalyst is generally nano‐sized, the preparation of nano‐NFS has not been achieved. Here, the micron‐sized NFS as an HER catalyst exhibits an outstanding overpotential of 6.5 mV at 90 °C and 10 mA cm^−2^ (Figure 5b) as well as an overpotential of 324.4 mV at 20 °C and 10 mA cm^−2^ (Figure 5c). Notably, the current exhibits a significant increase at zero voltage (Figure 5b), which implies that the micron‐sized NFS also has spontaneous catalysis activity ^[^ ^49^ ^]^ at high temperature (90 °C). And this spontaneous catalytic activation also leads to a sudden increase in the catalytic current when the voltage is applied (Figure 5b). As illustrated in Figure 5d, there is a fractional current density loss after continuous hydrogen generation at a static overpotential of 500 mV for 48 h. These properties have far exceeded those of the reported HER catalysts with the same size (**Table** **1**).^[^ ^50^, ^51^, ^52^, ^53^, ^54^, ^55^, ^56^, ^57^, ^58^, ^59^ ^]^ These results imply that the cheap (≈100 ＄ kg^−1^) NFS with micron size has the capability to be applied for large‐scale hydrogen production, and the components of S, Fe, and Na for the NFS are harmless to the environment.

**Table 1:** Comparison of catalytic performance of typical materials

Electrocatalysts	Size	Electrolyte	E (mV, vs RHE) at 10 mA cm^−2^	Long‐term stability	Ref
NiCoS‐120	Nanoflowers	0.5 m H_2_SO_4_	−382	–	[54]
NiCoS‐150			−232	20 h	
NiCoS‐180			−310	–	
NiCoS‐210			−358	–	
NiCoS‐240			−378	–	
ZnMoO_4_	Sub‐micron plates	0.5 m H_2_SO_4_	−1350	240 s	[53]
X (F, Br, I)‐RGO	Micron‐sized	0.5 m NaOH	−810	200 min	[55]
RGO	Micron‐sized		−880	200 min	
N‐GMT	1–2 µm inner voids	0.1 m KOH	−464	–	[56]
		6 m KOH	−426		
g‐C_3_N_4_ with N‐graphene	Micron‐sized	0.5 m H_2_SO_4_	−580	–	[57]
N,S‐graphene	Micron‐sized	0.5 m H_2_SO_4_	−310	–	[58]
S‐GNs	Micron‐sized	0.5 m H_2_SO_4_	−540	–	[59]
N‐pGr	Micron‐sized	0.5 m H_2_SO_4_	−484	–	[51]
P‐pGr			−409	–	
g‐C_3_N_4_@N‐pGr			−397	–	
g‐C_3_N_4_@ P‐pGr			−340	500 min	
NaFe_3_S_5_·2H_2_O	Micron‐sized	0.5 m H_2_SO_4_	−6.5 (90 °C)	–	This work
			−324.4 (20 °C)	48 h	

Ultimately, the adsorption model was established to simulate the major adsorption regions of ions between (0 0 1) crystal planes (Figure 5e), and the adsorption energies of different adsorption sites for lithium, sodium, and potassium ions within these regions were calculated (Figure 5f and Figure S4, Supporting Information). The results suggest that the storage of lithium, sodium, and potassium ions in the NFS is mainly concentrated in the gap between (0 0 1) crystal planes. As an example, a sodium‐ion battery of CR2032 was assembled with an NFS electrode. The Nyquist plots were collected from 0 to 10^5^ Hz on the sodium‐ion battery, and the results exhibit that this battery possesses the solution resistance (*R*_s_) of 172.4 Ω (Figure 5g), which represents that it has good electrical conductivity. Figure 5h illustrates the CV curves of this battery at a scanning rate of 0.1 V s^−1^ in the potential range of 0.0–6.0 V (vs Na^+^/Na), which suggests that NFS may be high‐voltage electrode materials.

## 3 Conclusions

In summary, we systematically introduced an embodiment of synthesizing NFS with stability in water–oxygen environments by the high‐pressure hydrothermal method, and proposed an analogous strategy to cicada molting that can be used to prepare unstable natural materials in water–oxygen conditions. The limitations of the high‐pressure hydrothermal method were broken through, which is of great scientific significance for its application to the synthesis of lunar minerals and Martian minerals in the future. Our works provide fundamental data of the NFS (Supporting Information) and a general route to synthesize newly discovered minerals based solely on their chemical formula. This opens up new possibilities for achieving novel applications and producing inexpensive (≈100 ＄ kg^−1^) three‐dimensional inorganic catalysts. Ultimately, the reduced cost renders the three‐dimensional NFS catalysts more appealing for practical applications.

## 4 Experimental Section

### Synthesis of Materials

Raw materials comprise 2 mmol ammonium iron (II) sulfate hexahydrate (Aladdin), 4 mmol sodium dodecyl sulfate (Aladdin) and 60 mL deionized water. Furthermore, the mixture of above raw materials with mixing completely was transferred into a Teflon reactor. The mixture underwent solvothermal reaction at 160 °C for 3 h in an oven, followed by natural cooling to room temperature. the product were washed and collected via the centrifugation method. Finally, the dried red powders were calcined at 400 °C for 4 h under nitrogen conditions to obtain NaFe_3_S_5_·2H_2_O crystals.

### Component and Morphology Characterization

The structure of the NaFe_3_S_5_·2H_2_O was characterized by X‐ray diffraction (XRD, Bruker D8 polycrystalline) with Cu‐K*α* radiation (*V* = 30 kV, *I* = 25 mA, *λ* = 1.5418 Å) over the 10° to 80° 2*θ* range. The chemical states of the samples were characterized by X‐ray photoelectron spectroscopy (XPS) with the Escalab 250Xi system at a pass energy of 150 eV (1 eV per step), using Al‐K*α* as the exciting X‐ray source. The spectra were calibrated with respect to the C 1s peak resulting from the adventitious hydrocarbon, which has an energy of 284.8 eV. The morphology and elemental distributions of the samples were obtained by S4800 scanning electron microscopy (SEM) with an energy dispersive X‐ray spectrometer (EDS), JEM‐2100 transmission electron microscope (TEM) and high‐resolution transmission electron microscopy (HRTEM; JEM‐2s100F, JEOL, Japan).

### Properties Calculations

Mechanical, optical and thermoelectric properties, tunable band gaps, geometry optimizations, band structures, and partial density of states were performed with the program package DMol^3^ and the Cambridge Sequential Total Energy Package (CASTEP) in the Material Studio 2020. For CASTEP program, a 3 × 4 × 3 k‐point grid was used, and the cutoff energy of the plane wave was 598.7 eV. The spin polarized Generalized Gradient Approximation (GGA) using the Perdewe–Burkee–Ernzerh (PBE) of exchange–correlation parameterization was adopted. Using Perdew–Wang (PW91) engenders the exchange correlation energy, and also the spin is considered. The maximum root‐mean‐square convergent tolerance of CASTEP program was less than 2.0 × 10^−6^ eV per atom. Geometry optimization was stopped when all relaxation forces of CASTEP program are less than 0.005 eV nm^−1^. For CASTEP program, the maximum displacement error was within 0.002 nm and the maximum stress was less than 0.1 GPa.

Fukui calculations were performed using Dmol^3^ module. The Fukui functions can be written by taking the finite difference approximations as:^[^ ^48^ ^]^

(3) $$f_{k}^{+}=q_{k}\left(N+1\right)-q_{k}\left(N\right)\;\left(\mathrm{for}\;\mathrm{nucleophilic}\;\;\mathrm{attack}\right)$$

(4) $$f_{k}^{-}=q_{k}\left(N\right)-q_{k}\left(N-1\right)\;\left(\mathrm{for}\;\mathrm{electrophilic}\;\;\mathrm{attack}\right)$$

(5) $$f_{k}^{0}=\frac{1}{2}\left[q_{k}\left(N+1\right)-q_{k}\left(N-1\right)\right]\;\left(\mathrm{for}\;\mathrm{radical}\;\;\mathrm{attack}\right)$$

where *q*_k_ is the gross charge of *k* atom, i.e.; the electronic density at a point r in space around the molecule. The *q*_k_(*N* + 1), *q*_k_(*N*), and *q*_k_(*N* − 1) were defined as the charge of the anionic, neutral and cationic species, respectively. Here Fukui functions were presented through the finite difference approximation using Hirshfeld population analysis.^[^ ^60^ ^]^

The propensity toward nucleophilic or electrophilic attacks can be more precisely predicted by the dual descriptor, which is pro‐posed by Morell et al.^[^ ^61^ ^]^ and defined as

(6) $$\Delta f\left(k\right)=f_{k}^{+}-f_{k}^{-}$$

### Elastic Constants Calculation

The elastic constants were calculated with the Forcite module in Materials Studio 2020 using the universal force field and current charges (Force field assigned) at ultra fine quality. The “atom based” electrostatic summation method and “atom based” van der Waals summation were chosen during all calculations. A 6 × 6 symmetric elastic constants matrix was calculated to describe fully the stress−strain relationship for the NaFe_3_S_5_ crystal. The bulk modulus, shear moduli, Young's modulus, and Poisson's ratio were calculated from the matrix based on the elasticity theory. The crystal anisotropy index was calculated as the ratio of the largest to the smallest Young's modulus.^[^ ^62^ ^]^

### Electrocatalytic Measurement

These measurements were carried out on a CHI 660E electrochemical workstation (Shanghai Chenhua Instrument Co., Ltd., China) within a three‐electrode cell in 0.5 m H_2_SO_4_ at room temperature. A glassy carbon electrode (GCE, a diameter of 3 mm) or a carbon paper (0.5 × 0.5 cm^−2^) covered by a thin catalyst film, a graphite rod and Ag/AgCl electrode (saturated KCl‐filled) were used as the working, counter electrode and reference electrodes, respectively. All potentials were referenced to the reversible hydrogen electrode (RHE) by the equation: *E* (vs RHE) = *E* (vs Ag/AgCl) + 0.197 + 0.059 × pH. To prepare the catalyst‐covered working electrode, a droplet of precursor solution (5 µL) was drop‐cast onto the surface of the polished GCE by a micro pipette (the loading for all catalysts was fixed at 0.283 mg cm^−2^), followed by plasma treatment at room temperature. Afterward, Nafion solution (5 µL, 0.05 wt%) was added onto the surface of the catalyst to ensure firm attachment during electrochemical measurements. Before starting the electrochemical experiments, the electrolyte was de‐aerated by purging with N_2_ for 30 min. Linear sweep voltammetry (LSV) curves were recorded at a scan rate of 5 mV s^−1^. All polarization curves were corrected with iR‐compensation. The long‐term stability was examined by recording a chronoamperometric curve at a constant overpotential of 500 mV for 48 h with iR‐compensation.

### Electrochemical Measurement

The working electrode for electrochemical properties was prepared by a mixture of the NFS, polyvinylidene fluoride (PVDF) and acetylene black (8:1:1, mass ratio). In the presence of trace 1‐Methyl‐2‐pyrrolidine (NMP), the above materials were mixed to produce a slurry. Then, it was evenly coated on aluminum foil, and dried at 80 °C overnights. Finally, a coin cell of CR 2032 was assembled in an argon‐filled glove box with metallic sodium as the counter electrode, a celgard 2400 membrane as the separator, a mixture of NaClO_4_ (1.0 mol L^−1^), ethylene carbonate (EC) and Diethyl carbonate (DEC) (1:1:1, volume ratio) as the electrolyte.

Cyclic voltammogram (CV) of the NFS/Na cell was tested by an electrochemical workstation (CHI 660E) in the range of 0.0–6.0 V (vs Na^+^/Na) at a scanning rate of 0.1 V s^−1^. The thin‐film electrode of NFS was used as a working electrode. The counter and reference electrodes were cylindrical stainless‐steel ingots. The area of all electrodes was 0.785 cm^2^. AC impedance spectroscopy of the coin cell was performed in the frequency range from 0.0001 Hz to 100 kHz. All the electrochemical measurements were investigated in a dry air atmosphere at room temperature.

## Conflict of Interest

The authors declare no conflict of interest.

## Author Contributions

H.D. and W.D. contributed equally to this work. H.Q. and W.Q. conceived and designed the project. H.D., Y.K., and Y.Y. performed all data collection. H.D. and W.Q. conducted the analysis of experimental data and simulations. H.Q. and W.Q. wrote the manuscript. Other authors corrected the manuscript.

## Supporting information

Supporting Information

Supporting Information

Supplemental Video 1

## Data Availability

Research data are not shared.
