# Supplementary material for: A Strategy Inspired by the Cicada Shedding Its Skin for Synthesizing the Natural Material NaFe3S5·2H2O
Source: Adv Sci (Weinh). 2023 May 10;10(21):2301324. doi: 10.1002/advs.202301324 (PMC10375171; doi:10.1002/advs.202301324)
Supplement: Supplementary file 1 — Supporting Information [file ADVS-10-2301324-s003.pdf]

ADVANCED SCIENCE
Open Access

Supporting Information

for *Adv. Sci.*, DOI 10.1002/advs.202301324

A Strategy Inspired by the Cicada Shedding Its Skin for Synthesizing the Natural Material $NaFe_3S_5 \cdot 2H_2O$

Hanqing Dai, Wenqing Dai, Yuanyuan Chen, Yukun Yan, Guangzheng Zuo, Zhe Hu, Jinxin Wei, Wenjie Zhou, Wanlu Zhang, Wei Wei*, Guoqi Zhang* and Ruiqian Guo**

A strategy inspired by the cicada sloughing its skin for synthesizing the natural material $NaFe_3S_5 \cdot 2H_2O$

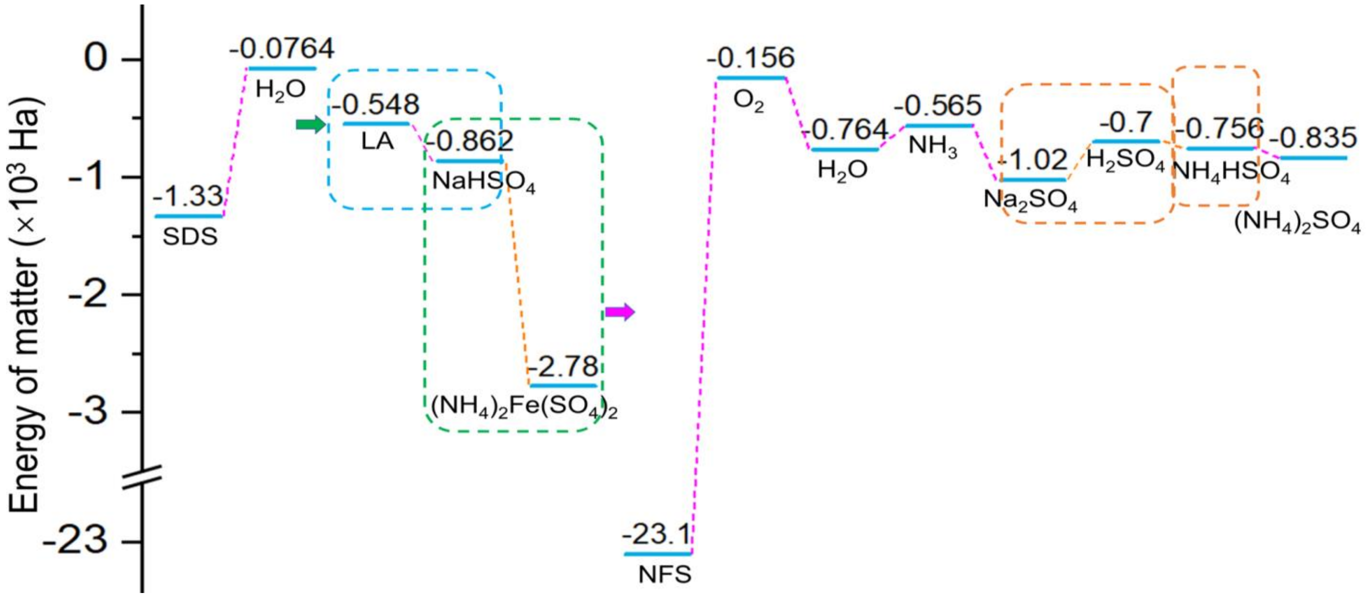

Figure S1. Energies of reactants and products were calculated by the DFT simulation.

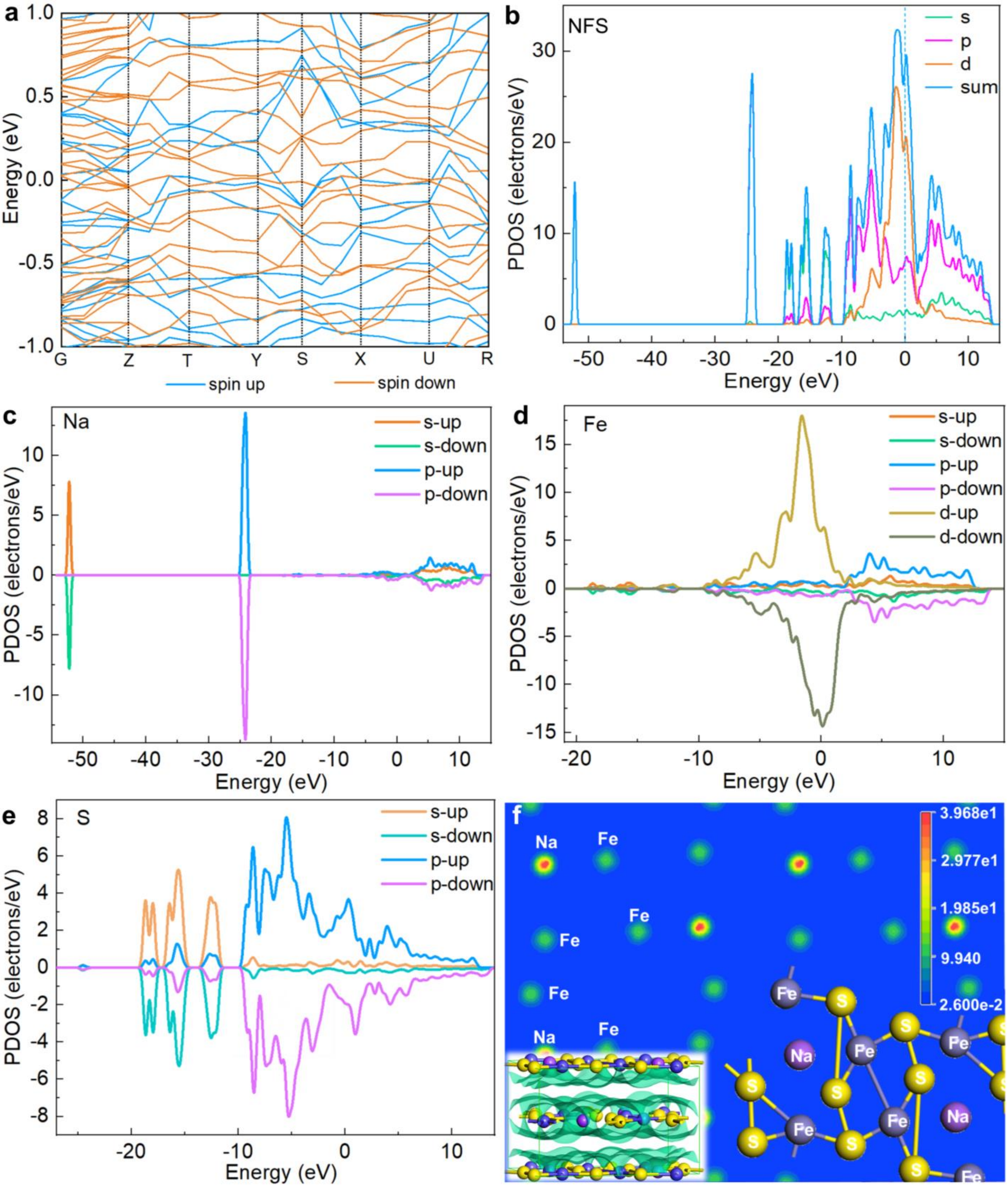

Figure S2. The electronic structure of the $NaFe_3S_5$ crystal model. a, Total band structures. **b**, Spin partial density of states. **c-e**, Partial density of states of the $NaFe_3S_5$ with Na, Fe and S, respectively. **f**, Electric charge density difference of the crystal plane (0 0 1) of the NFS attached with a local enlargement map.

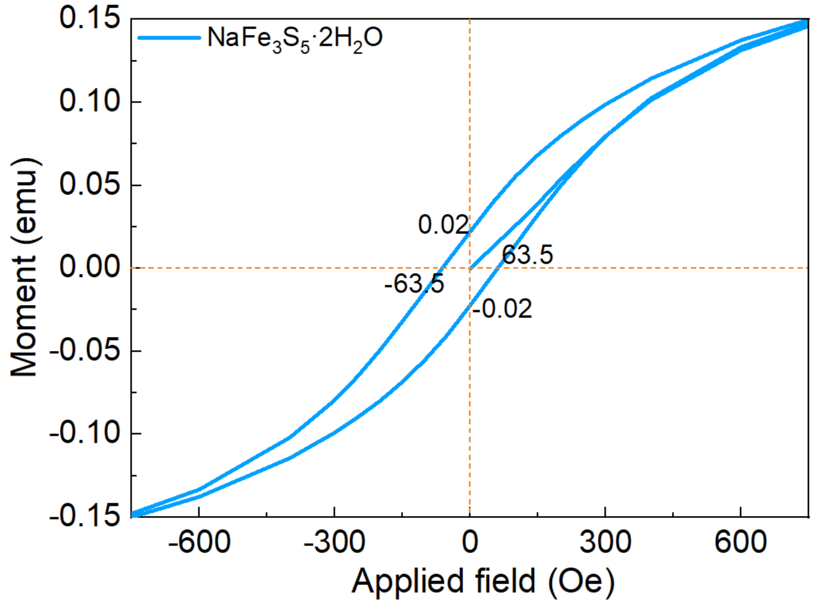

Figure S3. The magnetization curve of the $NaFe_3S_5 \cdot 2H_2O$.

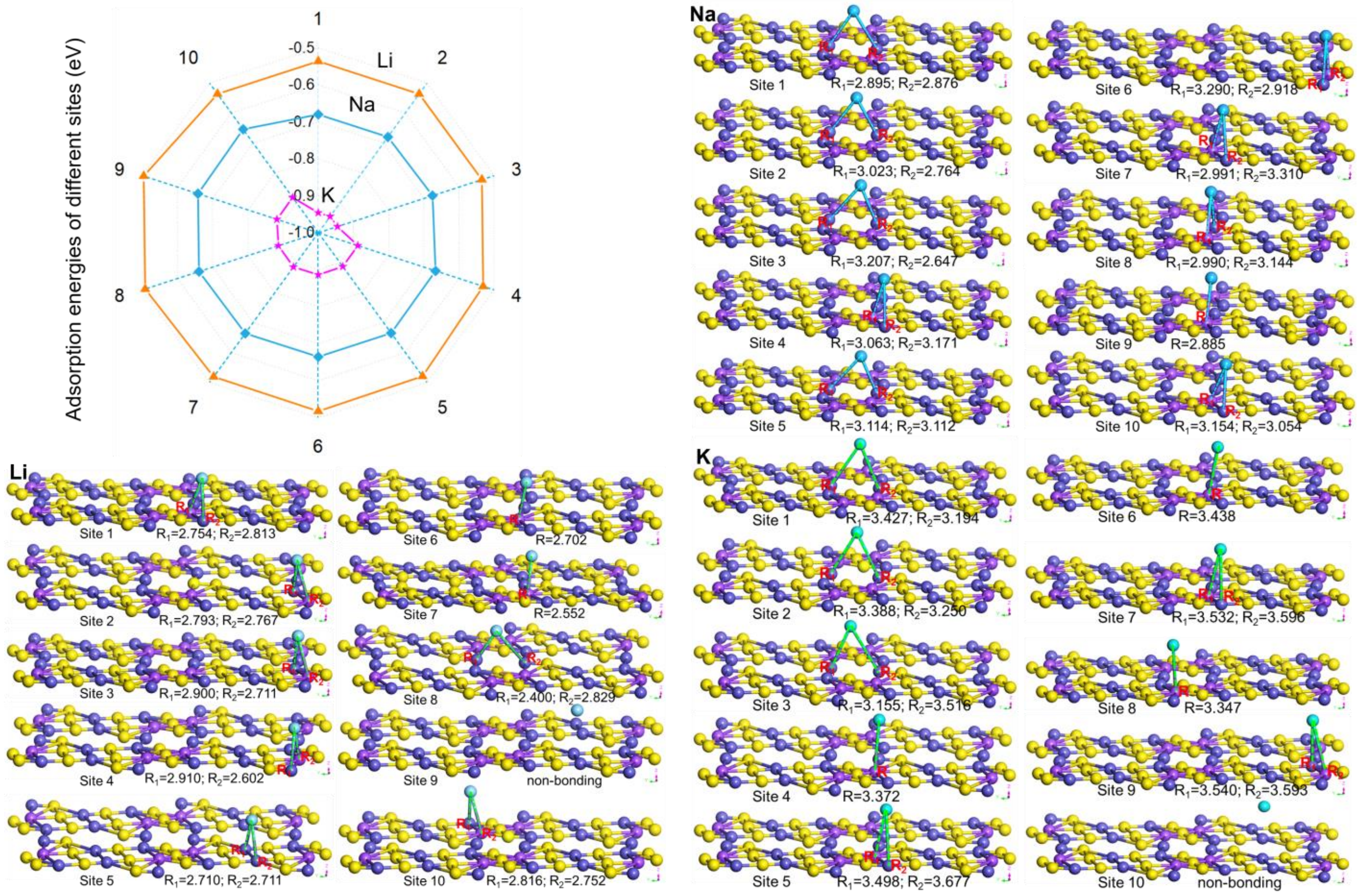

Figure S4. Adsorption energies (unit: eV) and bond distances (unit: Å) of different adsorption sites for lithium, sodium and potassium ions in the adsorption regions between (0 0 1) crystal planes of the $NaFe_3S_5$ crystal model.

Table S1| Fukui functions and dual descriptor of the $NaFe_3S_5$ crystal model.

Atom	f^0	f^+	f^-	Δf
S (1)	0.041	0.023	0.059	-0.036
S (2)	0.041	0.023	0.059	-0.036
S (3)	0.025	0.019	0.031	-0.012
S (4)	0.023	0.018	0.028	-0.01
S (5)	0.031	0.019	0.043	-0.024
S (6)	0.037	0.022	0.051	-0.029
S (7)	0.034	0.022	0.047	-0.025
Fe (8)	0.069	0.043	0.095	-0.052
Fe (9)	0.054	0.044	0.065	-0.021
Na (10)	0.069	0.038	0.099	-0.061
S (11)	0.025	0.019	0.031	-0.012
S (12)	0.023	0.018	0.028	-0.01
S (13)	0.031	0.019	0.043	-0.024
S (14)	0.037	0.022	0.051	-0.029
S (15)	0.034	0.022	0.047	-0.025
Fe (16)	0.069	0.043	0.095	-0.052
Fe (17)	0.054	0.044	0.065	-0.021
Na (18)	0.069	0.038	0.099	-0.061
S (19)	0.025	0.019	0.031	-0.012
S (20)	0.023	0.018	0.028	-0.01
S (21)	0.031	0.019	0.043	-0.024
S (22)	0.037	0.022	0.051	-0.029
S (23)	0.034	0.022	0.047	-0.025
Fe (24)	0.069	0.043	0.095	-0.052
Fe (25)	0.054	0.044	0.065	-0.021
Na (26)	0.069	0.038	0.099	-0.061
S (27)	0.025	0.019	0.031	-0.012
S (28)	0.023	0.018	0.028	-0.01
S (29)	0.031	0.019	0.043	-0.024
S (30)	0.037	0.022	0.051	-0.029
S (31)	0.034	0.022	0.047	-0.025
Fe (32)	0.069	0.043	0.095	-0.052
Fe (33)	0.054	0.044	0.065	-0.021
Na (34)	0.069	0.038	0.099	-0.061

Fundamental data of the $NaFe_3S_5$ crystal

Cell vectors

	14.00098086007751	0.00000000000000	0.00000000000000
	0.00000000000000	18.67238384106166	0.00000000000000
	0.00000000000000	0.00000000000000	12.17172597108371

Coordinates

S	0.00000000000000	9.33619192053083	6.08586298554186
S	7.00049043003875	0.00000000000000	0.00000000000000
S	9.85669047258223	0.93361920528117	0.00000000000000
S	4.24229724406718	11.72625744336003	0.00000000000000
S	5.46038233511926	5.97516269496918	0.00000000000000
S	1.27408923181089	5.62038751915202	0.00000000000000
Fe	0.74205197613548	2.91289193967686	0.00000000000000
Fe	3.75226296309735	14.99392372548482	0.00000000000000
Fe	4.73233152314729	8.88805491243577	0.00000000000000
Na	5.82440815873471	2.24068600990479	0.00000000000000
S	4.14429038749527	17.73876463578049	0.00000000000000
S	9.75868361601032	6.94612639770163	0.00000000000000
S	8.54059852495825	12.69722114609248	0.00000000000000
S	12.72689162826662	13.05199632190964	0.00000000000000
Fe	13.25892888394203	15.75949190138481	0.00000000000000
Fe	10.24871789698015	3.67846011557684	0.00000000000000
Fe	9.26864933693021	9.78432892862589	0.00000000000000
Na	8.17657270134279	16.43169783115687	0.00000000000000
S	11.14478081753403	10.26981112581200	6.08586298554186
S	2.75819318597157	2.39006552282920	6.08586298554186
S	1.54010809491949	15.31135461550001	6.08586298554186
S	5.72640119822787	14.95657943968286	6.08586298554186
Fe	6.25843845390328	12.24908386020769	6.08586298554186
Fe	3.24822746694140	5.65773180495399	6.08586298554186
Fe	2.26815890689146	18.22424683296660	6.08586298554186
Na	1.17608227130404	11.57687793043563	6.08586298554186
S	2.85620004254348	8.40257271524966	6.08586298554186
S	11.24278767410594	16.28231831823246	6.08586298554186
S	12.46087276515801	3.36102922556165	6.08586298554186
S	8.27457966184964	3.71580440137881	6.08586298554186
Fe	7.74254240617423	6.42329998085398	6.08586298554186
Fe	10.75275339313610	13.01465203610767	6.08586298554186
Fe	11.73282195318605	0.44813700809506	6.08586298554186
Na	12.82489858877347	7.09550591062604	6.08586298554186

Sodium nbas= 1, z= 11, nrfn= 7, rcut= 9.83, e_ref= -0.014276 Ha

rcore= 0.00 zval= 11.00 11.00
n=1 L=0 occ= 2.00 e= -37.925653Ha -1032.0100eV
n=2 L=0 occ= 2.00 e= -2.060006Ha -56.0556eV
n=2 L=1 occ= 6.00 e= -1.038710Ha -28.2648eV
n=3 L=0 occ= 1.00 e= -0.083300Ha -2.2667eV
n=3 L=0 occ= 0.00 e= -0.268943Ha -7.3183eV
n=3 L=1 occ= 0.00 e= -0.136566Ha -3.7161eV
n=3 L=2 occ= 0.00 e= -0.888739Ha -24.1838eV
Sulfur nbas= 2, z= 16, nrfn= 8, rcut= 9.83, e_ref= -0.031417 Ha
rcore= 0.00 zval= 16.00 16.00
n=1 L=0 occ= 2.00 e= -88.141883Ha -2398.4637eV
n=2 L=0 occ= 2.00 e= -7.734294Ha -210.4609eV
n=2 L=1 occ= 6.00 e= -5.756585Ha -156.6447eV
n=3 L=0 occ= 2.00 e= -0.630454Ha -17.1555eV
n=3 L=1 occ= 4.00 e= -0.258477Ha -7.0335eV
n=3 L=0 occ= 0.00 e= -1.445057Ha -39.3220eV
n=3 L=1 occ= 0.00 e= -1.031084Ha -28.0572eV
n=3 L=2 occ= 0.00 e= -0.484479Ha -13.1833eV
Iron nbas= 3, z= 26, nrfn= 10, rcut= 9.83, e_ref= -0.137855 Ha
rcore= 0.00 zval= 26.00 26.00
n=1 L=0 occ= 2.00 e= -254.842923Ha -6934.6317eV
n=2 L=0 occ= 2.00 e= -29.630891Ha -806.2979eV
n=2 L=1 occ= 6.00 e= -25.576309Ha -695.9671eV
n=3 L=0 occ= 2.00 e= -3.372905Ha -91.7815eV
n=3 L=1 occ= 6.00 e= -2.187295Ha -59.5194eV
n=3 L=2 occ= 6.00 e= -0.282852Ha -7.6968eV
n=4 L=0 occ= 2.00 e= -0.188063Ha -5.1175eV
n=3 L=2 occ= 0.00 e= -1.448006Ha -39.4023eV
n=4 L=0 occ= 0.00 e= -0.900065Ha -24.4920eV
n=4 L=1 occ= 0.00 e= -0.654625Ha -17.8133eV

Charge partitioning by Hirshfeld method:

S	1 charge	-0.0585
S	2 charge	-0.0585
S	3 charge	-0.0319
S	4 charge	-0.0513
S	5 charge	-0.1562
S	6 charge	0.0108
S	7 charge	0.0334
Fe	8 charge	0.0974
Fe	9 charge	0.0676
Na	10 charge	0.0846
S	11 charge	-0.0319
S	12 charge	-0.0513

S	13 charge	-0.1562
S	14 charge	0.0108
S	15 charge	0.0334
Fe	16 charge	0.0974
Fe	17 charge	0.0676
Na	18 charge	0.0846
S	19 charge	-0.0319
S	20 charge	-0.0513
S	21 charge	-0.1562
S	22 charge	0.0108
S	23 charge	0.0334
Fe	24 charge	0.0974
Fe	25 charge	0.0676
Na	26 charge	0.0846
S	27 charge	-0.0319
S	28 charge	-0.0513
S	29 charge	-0.1562
S	30 charge	0.0108
S	31 charge	0.0334
Fe	32 charge	0.0974
Fe	33 charge	0.0676
Na	34 charge	0.0846

Mulliken populations summed for each angular momentum shell:

1 S	charge=		0.770
	s	5.218	
	p	8.572	
	d	1.440	
2 S	charge=		0.770
	s	5.218	
	p	8.572	
	d	1.440	
3 S	charge=		0.685
	s	5.276	
	p	8.532	
	d	1.508	
4 S	charge=		0.800
	s	5.231	
	p	8.454	
	d	1.514	
5 S	charge=		0.949
	s	5.226	
	p	8.356	

	d	1.469	
6 S	charge=		0.655
	s	5.287	
	p	8.575	
	d	1.483	
7 S	charge=		0.533
	s	5.283	
	p	8.680	
	d	1.504	
8 Fe	charge=		-0.564
	s	6.873	
	p	13.366	
	d	6.325	
9 Fe	charge=		-0.723
	s	6.863	
	p	13.572	
	d	6.289	
10 Na	charge=		-1.170
	s	5.016	
	p	6.795	
	d	0.358	
11 S	charge=		0.685
	s	5.276	
	p	8.532	
	d	1.508	
12 S	charge=		0.800
	s	5.231	
	p	8.454	
	d	1.514	
13 S	charge=		0.949
	s	5.226	
	p	8.356	
	d	1.469	
14 S	charge=		0.655
	s	5.287	
	p	8.575	
	d	1.483	
15 S	charge=		0.533
	s	5.283	
	p	8.680	
	d	1.504	
16 Fe	charge=		-0.564
	s	6.873	
	p	13.366	
	d	6.325	

17 Fe	charge=	-0.723
	s 6.863	
	p 13.572	
	d 6.289	
18 Na	charge=	-1.170
	s 5.016	
	p 6.795	
	d 0.358	
19 S	charge=	0.685
	s 5.276	
	p 8.532	
	d 1.508	
20 S	charge=	0.800
	s 5.231	
	p 8.454	
	d 1.514	
21 S	charge=	0.949
	s 5.226	
	p 8.356	
	d 1.469	
22 S	charge=	0.655
	s 5.287	
	p 8.575	
	d 1.483	
23 S	charge=	0.533
	s 5.283	
	p 8.680	
	d 1.504	
24 Fe	charge=	-0.564
	s 6.873	
	p 13.366	
	d 6.325	
25 Fe	charge=	-0.723
	s 6.863	
	p 13.572	
	d 6.289	
26 Na	charge=	-1.170
	s 5.016	
	p 6.795	
	d 0.358	
27 S	charge=	0.685
	s 5.276	
	p 8.532	
	d 1.508	
28 S	charge=	0.800

	s	5.231	
	p	8.454	
	d	1.514	
29 S	charge=		0.949
	s	5.226	
	p	8.356	
	d	1.469	
30 S	charge=		0.655
	s	5.287	
	p	8.575	
	d	1.483	
31 S	charge=		0.533
	s	5.283	
	p	8.680	
	d	1.504	
32 Fe	charge=		-0.564
	s	6.873	
	p	13.366	
	d	6.325	
33 Fe	charge=		-0.723
	s	6.863	
	p	13.572	
	d	6.289	
34 Na	charge=		-1.170
	s	5.016	
	p	6.795	
	d	0.358	
